# Live IVUS-guided wiring in percutaneous coronary intervention: a case report

**DOI:** 10.1093/ehjcr/ytag111

**Published:** 2026-02-12

**Authors:** Rithik Mohan Singh Sindhi, Divyesh Sharma, Jack Andrews

**Affiliations:** Department of Cardiology, Western Health & Social Care Trust, Glenshane Road, Londonderry BT47 6SB, Northern Ireland; Department of Cardiology, Western Health & Social Care Trust, Glenshane Road, Londonderry BT47 6SB, Northern Ireland; Department of Cardiology, Western Health & Social Care Trust, Glenshane Road, Londonderry BT47 6SB, Northern Ireland

**Keywords:** Case report, Real-time intravascular ultrasound (RT-IVUS), Intravascular ultrasound (IVUS), Bifurcation lesion, Chronic total occlusion, Dual lumen microcatheter

## Abstract

**Background:**

Wiring in bifurcation percutaneous coronary intervention can be limited by severe angulation, adverse plaque biasing, and ostial ambiguity. Real-time intravascular ultrasound (RT-IVUS) guidance can augment wire manipulation when standard techniques fail.

**Case summary:**

A 49-year-old man with exertional angina had a critical lesion left anterior descending (LAD)-2nd diagonal bifurcation lesion (Medina 1,1,0) with TIMI 2 flow. Standard wiring (acutely angled wires, dual lumen, and angled microcatheters) repeatedly biased into the diagonal. Under RT-IVUS, the wire was redirected into the true lumen of mid-LAD, enabling lesion crossing, pre-dilatation, and implantation of a 3.5 × 26 mm^2^ DES with post-dilatation. Final IVUS showed optimal expansion (MSA 9 mm^2^). The patient was discharged same day; at 3 months, he remained asymptomatic with a patent stent.

**Conclusion:**

RT-IVUS can overcome wiring failure in complex bifurcations by providing live intravascular guidance augmenting wire passage into true lumen, supporting safe, efficient revascularization.

Learning points

- Clinical: RT-IVUS should be considered when conventional wiring strategies, including angled or dual-lumen microcatheters, fail in complex bifurcation lesions.
- Technical: A 7 Fr guiding catheter is typically required to accommodate an IVUS catheter and guidewire within a microcatheter.
- Practical: Severe calcification or marked vessel tortuosity may limit successful positioning of the IVUS catheter, reducing feasibility in some cases.

## Introduction

Percutaneous coronary intervention (PCI) is an important treatment of obstructive coronary artery disease, especially in those in whom CABG targets are poor or surgical risk is prohibitively high. The frequency of complex PCI [bifurcation lesions and chronic total occlusions (CTOs)] in this cohort has increased dramatically in recent years.^1,2^ While traditional techniques can achieve successful outcomes, they often encounter challenges when dealing with difficult anatomy or ambiguous lesion morphology. In such cases, intravascular ultrasound (IVUS) guidance offers significant advantages by providing real-time, high-resolution internal imaging of coronary anatomy and plaque morphology.^3,4^

This case report presents a 49-year-old male with a critical left anterior descending (LAD) bifurcation lesion, demonstrating the successful use of real-time intravascular ultrasound (RT-IVUS)-guided wiring to navigate complex coronary anatomy. IVUS not only facilitated accurate wire placement but also allowed for optimal lesion assessment, leading to improved procedural outcomes and patient safety. The growing role of IVUS in PCI underscores its value in overcoming the limitations of conventional angiographic guidance, enhancing procedural precision, and ultimately improving clinical outcomes. This case highlights the potential of IVUS to become an indispensable tool in the interventional cardiologist's armamentarium before sizing stents or deciding on calcium modification.

## Summary figure

**Figure:**
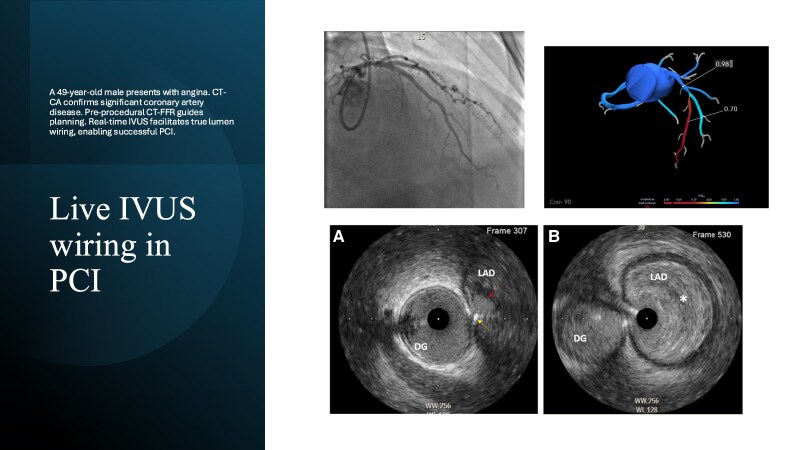


## Case presentation

### Patient information

A 49-year-old gentleman with a history of asthma and hyperlipidaemia presented with exertional angina. The patient had no prior history of myocardial infarction or previous coronary interventions. He had been experiencing progressive exertional chest discomfort over the past 6 months, which limited his daily activities. A CTCA performed in September 2024 revealed a significant stenosis in the proximal LAD, prompting invasive coronary angiography.

### Diagnostic assessment

The pre-procedure echocardiography showed normal left ventricular size and function, with a preserved ejection fraction. Coronary angiography revealed several key findings. The Left Main Stem appeared normal. The LAD artery exhibited a critical LAD bifurcation lesion classified as Medina 1,1,0 with TIMI 2 flow. The left circumflex showed minor plaque burden with no significant stenosis. The right coronary artery was dominant and free of disease.

Clinical assessment and labs: Physical examination was normal. Baseline laboratory values: troponin 6 ng/L, HbA1c 32 mmol/mol, NT-proBNP 17 ng/L. Lipid profile: total cholesterol 6.4 mmol/L, LDL-cholesterol 4.6 mmol/L.

**Figure 1:**
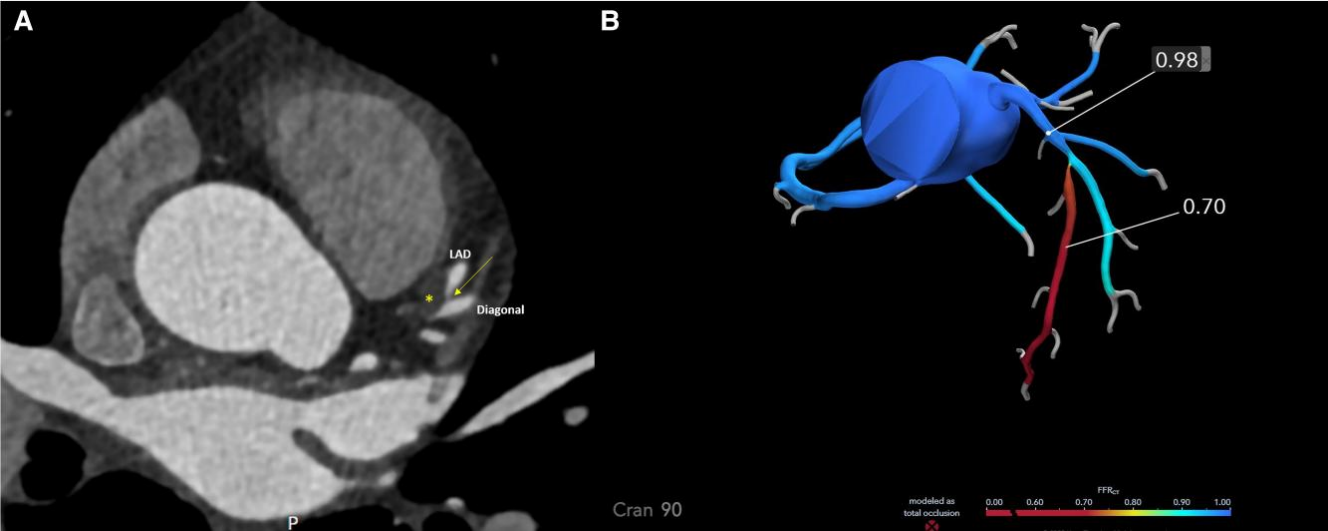
(*A*) Axial CTCA at the level of the bifurcation of the left anterior descending with the large first diagonal branch. The yellow asterisk (*) shows the low-density lipid rich plaque within the proximal and mid-left anterior descending with medina classification of 1,1,0. The yellow arrow shows the very narrow ostium of the mid-left anterior descending. (*B*) CT-derived fractional flow reserve demonstrates a proximal value of 0.98 falling to 0.70 distally across the mid-left anterior descending lesion, confirming haemodynamic significance.

To aid in procedural planning, a CT-derived fractional flow reserve (CT-FFR) analysis was performed. The CT-FFR map demonstrated a significant pressure drop across the mid-LAD lesion, with values decreasing from 0.98 proximally to 0.70 distally, confirming non-invasive haemodynamic significance. This assessment supported the decision for revascularization and informed the strategy for PCI. Furthermore, the plaque appeared soft and non-calcified informing the operators that balloon therapy was likely sufficient prior to stent implantation (*Figure 1*).

### Procedure

**Figure 2:**
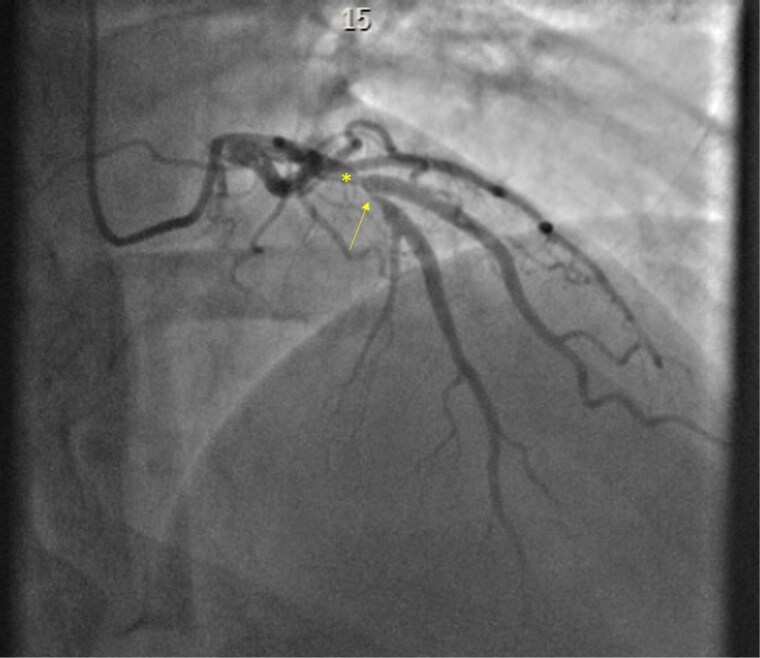
Coronary angiography in the RAO cranial view. Note the severe disease proximal to the bifurcation with the first diagonal branch (yellow asterisk) as well as the critical ostial stenosis in the mid-left anterior descending (yellow arrow). The ostium of the diagonal branch is free of disease, making it a medina 1,1,0 classification.

PCI was performed via a 7 Fr right radial access using an EBU3.5 guide catheter. The initial approach involved wiring the diagonal branch using a Sion Blue wire, while multiple attempts were made to cross the LAD lesion using a highly angulated workhorse wire (BMW), a polymer jacketed Fielder XT-A, and Sion black wire. These initial attempts were unsuccessful due to severe angulation and the propensity of the wire to deflect away from the true lumen due to adverse plaque bias and prolapse towards the diagonal branch (*Figure 2*). Given the failure of conventional wiring techniques, the strategy shifted to more aggressive shaping of the wire tip to optimize directional control. Despite this, wiring remained unsuccessful due to the complex angulation and ostial ambiguity. A dual-lumen microcatheter (Sasuke) was introduced, but this approach was also ineffective. Subsequently, an angled 90-degree Supercross microcatheter was employed, but significant plaque burden continued to bias the wire away from the desired trajectory.

**Figure 3:**
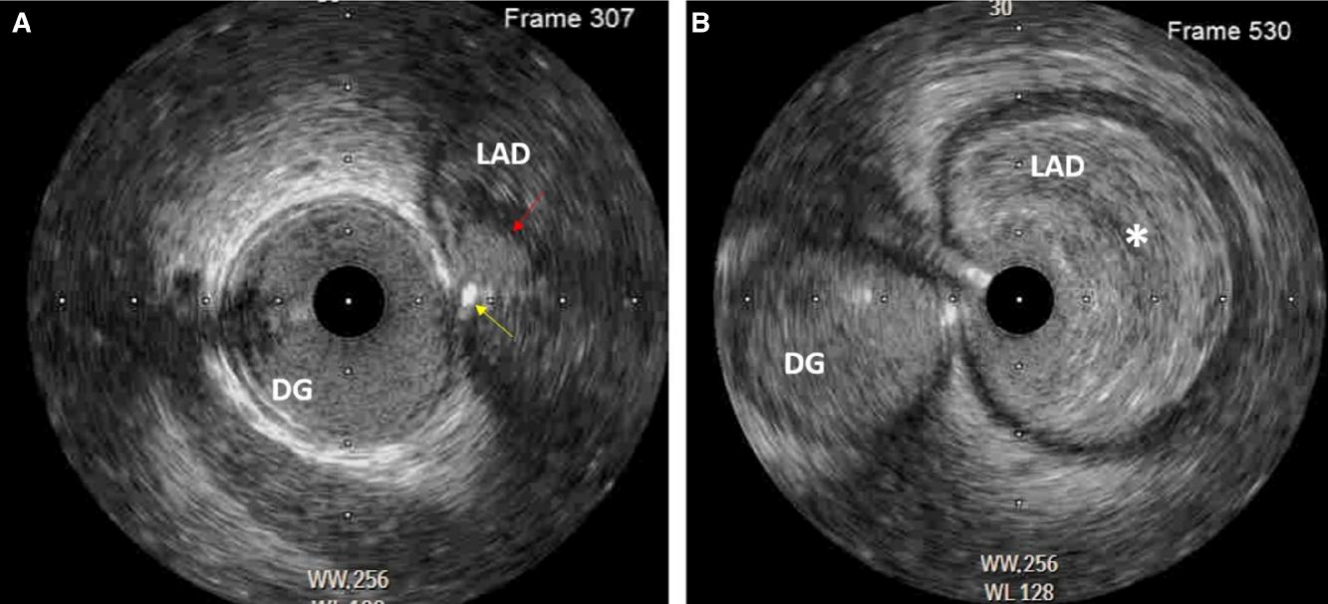
Panel (*A*) shows the intravascular ultrasound catheter at the level of the left anterior descending bifurcation, the mid-left anterior descending, and diagonal branches. The red arrow shows the small orifice of the mid-left anterior descending ostium. Using live intravascular ultrasound guided imaging, we were able to successfully wire the left anterior descending (yellow arrow). Panel (*B*) shows the intravascular ultrasound catheter within the mid-left anterior descending. Note the large burden of soft lipid rich plaque (*).

RT-IVUS technique: A workhorse wire was positioned in the diagonal branch, over which an IVUS catheter was placed. The IVUS probe was then manually pulled back to localize the ostium of the LAD in real time. This allowed the operator to appreciate how small the ostium of the mid-LAD was and why conventional wiring techniques had proved ineffectual. A brief angiographic acquisition confirmed the IVUS probe position (*Figure 3*) (see Supplementary material online, video). Under RT-IVUS visualization, the operator was able to guide the wire with a high degree of precision into the LAD lumen, allowing subsequent lesion preparation and stent delivery.

IVUS imaging provided critical information regarding the lesion's morphology, vessel dimensions, and optimal wire trajectory. Under IVUS guidance, successful lesion crossing was achieved, followed by pre-dilatation using a 2.5 mm semi-compliant and 3.5 mm non-compliant balloon. Stenting was performed using a 3.5 × 26 mm^2^ drug-eluting stent, followed by post-dilatation with a 4.5 mm non-compliant balloon. Final IVUS assessment confirmed a minimal stent area of 9 mm^2^ with excellent stent expansion and apposition.

Procedural metrics: Total procedure duration was 66 min. Fluoroscopy time was 10 min. Recorded air kerma 803 mGy. Total contrast volume 120 mL. Stent sizing and optimization were guided by IVUS-derived vessel measurements.

### Outcome and follow-up

The patient was discharged on the same day with dual antiplatelet therapy (aspirin and a P2Y12 inhibitor) and lifestyle modification guidance. No complications were reported at follow-up, and repeat imaging at 3 months confirmed stent patency and restoration of normal coronary flow. The patient remained asymptomatic and had resumed normal physical activities without recurrence of anginal symptoms.

## Discussion

**Figure 4:**
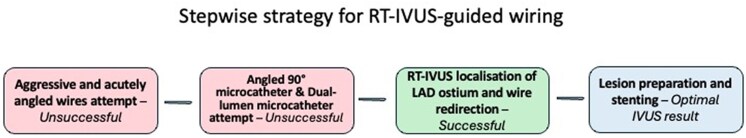
Schematic summary of the stepwise wiring strategy.

This case highlights the utility of RT-IVUS-guidance in wiring complex bifurcation lesions challenged with severe angulation and adverse biasing towards the side branch. Fluoroscopy provides two-dimensional visualization of a three-dimensional structure, making precise axial alignment with a small diseased LAD ostium challenging. This explains why our initial approach with an angled 90° SuperCross microcatheter and a dual-lumen microcatheter failed. The side port on a dual-lumen microcatheter may lie anywhere on a 360° axial spectrum, with minimal operator manoeuvrability. Reverse wiring can help in sharply angulated vessels, but heavy proximal plaque can bias the wire away from the target lumen, limiting success. optical coherence tomography (OCT) would delineate the LAD ostium and plaque composition, but stationary imaging typically requires continuous contrast or saline flushing; this increases contrast load and with prolonged saline, risks QT prolongation and ventricular arrhythmia. In this case, RT-IVUS provided continuous intraluminal visualization without repeated contrast injections and allowed tip-by-tip redirection into the LAD ostium, converting failure to success (*Figure 4*).

Prior reports have described RT-IVUS roles in CTO-PCI such as cap identification and puncture, discrimination of intimal vs. subintimal wire position, and tip-directed antegrade dissection-re-entry principles that we adapted for a complex bifurcation rather than re-entry of the true lumen in CTO.

Standard angiography alone may not provide sufficient resolution to delineate luminal entry points in cases with significant ostial ambiguity. IVUS enhances procedural efficacy by adding an alternative imaging modality that allows for improved wiring precision. Careful co-ordination is required during simultaneous wire and IVUS manipulation at a tight ostium to minimize the risk of vessel injury and occlusion.

Limitations: RT-IVUS wiring is technically demanding and highly dependent on operator expertise and experience with intravascular imaging interpretation. The technique generally requires a 7 Fr guiding catheter to allow simultaneous manipulation of the IVUS catheter and guidewire, which may limit its use in smaller radial arteries. In addition, advancing an IVUS catheter may be challenging in the presence of severe proximal calcification or marked vessel tortuosity, potentially precluding its application in some anatomies. Finally, this report represents a single case, and the generalisability of this approach cannot be assumed without further evaluation in larger series.

One of the key advantages of IVUS over traditional angiography is its ability to provide real-time cross-sectional images of the vessel lumen, facilitating a more precise assessment of lesion morphology.^5^ This is particularly relevant in bifurcation lesions, where understanding the plaque composition, distribution, and vessel remodelling is essential for selecting an appropriate treatment strategy.^6^ Moreover, IVUS enables accurate measurement of luminal dimensions, which aids in selecting the correct stent size and optimizing deployment techniques.^6^ Studies have shown that IVUS-guided stenting results in larger minimal stent areas, which are associated with reduced rates of restenosis and target lesion failure.^7^ Furthermore, in CTOs PCI, the role of RT-IVUS in identifying the proximal cap and its composition (calcified vs. non-calcified) informs on accurate guidewire selection and entry, thereby minimizing the risk of subintimal passage and potential vessel perforation.^8^

Additionally, IVUS reduces the need for contrast-based imaging, making PCI safer for patients at risk of contrast-induced nephropathy.^9,10^ Future advancements, such as artificial intelligence-assisted IVUS interpretation and hybrid imaging techniques integrating IVUS and OCT, are expected to further refine lesion assessment and procedural strategies.^11–13^

This technique requires a suitably sized guiding catheter, typically a 7 Fr to accommodate both IVUS and microcatheters.

## Conclusion

This case underscores the importance of IVUS guidance in PCI, particularly when wiring challenging bifurcation lesions with severe angulation and adverse plaque bias where the use of adjunctive angled and dual-lumen microcatheters alone is unsuccessful. IVUS enables real-time visualization, guides wire positioning, and ensures adequate plaque modification, stent sizing, and improved clinical outcomes. The integration of advanced imaging modalities into routine PCI practice should be encouraged to enhance procedural efficacy and patient safety. With continued technological advancements and greater operator experience, IVUS-guided PCI is expected to play an increasingly central role in interventional cardiology.

## Lead author biography


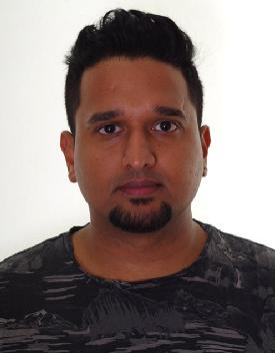


Rithik Mohan Singh Sindhi is a Cardiology Registrar currently in training at the Northern Ireland Deanery. He has a strong interest in interventional cardiology, with a particular focus on complex PCI and the application of advanced intracoronary imaging techniques such as IVUS and OCT. He is actively engaged in academic writing and case-based cardiovascular education. Dr Mohan is passionate about improving procedural precision and patient outcomes through imaging-guided interventions. He aims to pursue further subspecialty training in interventional cardiology and contribute to the advancement of evidence-based cardiovascular practice.

## Supplementary Material

ytag111_Supplementary_Data

## Data Availability

All data underlying this article are available within the published manuscript. Additional procedural materials (including angiographic and IVUS video recordings) are available from the corresponding author upon reasonable request.
